# Expanding Diversity and Common Goal of Regulatory T and B Cells. I: Origin, Phenotype, Mechanisms

**DOI:** 10.1007/s00005-017-0469-3

**Published:** 2017-05-05

**Authors:** Katarzyna Bocian, Ewelina Kiernozek, Joanna Domagała-Kulawik, Grażyna Korczak-Kowalska, Anna Stelmaszczyk-Emmel, Nadzieja Drela

**Affiliations:** 1 Department of Immunology, Faculty of Biology, University of Warsaw, Warsaw, Poland; 2 Department Pneumonology, Medical University of Warsaw, Warsaw, Poland; 3 Department of Laboratory Diagnostics and Clinical Immunology of Developmental Age, Medical University of Warsaw, Warsaw, Poland; 4 Department of Clinical Immunology, Transplantation Institute, Medical University of Warsaw, Warsaw, Poland

**Keywords:** Regulatory B cell, Regulatory T cell, Immunosuppression, Epigenetics

## Abstract

Immunosuppressive activity of regulatory T and B cells is critical to limit autoimmunity, excessive inflammation, and pathological immune response to conventional antigens or allergens. Both types of regulatory cells are intensively investigated, however, their development and mechanisms of action are still not completely understood. Both T and B regulatory cells represent highly differentiated populations in terms of phenotypes and origin, however, they use similar mechanisms of action. The most investigated CD4^+^CD25^+^ regulatory T cells are characterized by the expression of Foxp3^+^ transcription factor, which is not sufficient to maintain their lineage stability and suppressive function. Currently, it is considered that specific epigenetic changes are critical for defining regulatory T cell stability in the context of their suppressive function. It is not yet known if similar epigenetic regulation determines development, lineage stability, and function of regulatory B cells. Phenotype diversity, confirmed or hypothetical developmental pathways, multiple mechanisms of action, and role of epigenetic changes in these processes are the subject of this review.

## Introduction

The immune system is indispensable for maintaining the integrity of an organism by protecting it from attacks against healthy self-cells, the elimination of aged and cancer cells, the inhibition of an excessive harmful response to conventional antigens or environmental agents (mainly allergens), and protection from pathogenic infections. The pro- and anti-inflammatory functions of the immune system are strictly regulated. The following inhibitory populations of immune cells have been currently recognized: regulatory T cells, Tregs (Benoist and Mathis 2012; Ohkura et al. 2013), regulatory B cells, Bregs (DiLillo et al. 2010; Mauri 2010), and regulatory myeloid cells (Coombes and Powrie 2008; Varol et al. 2010). Nevertheless, the main scientific interest is still focused on Treg cells of the CD4^+^CD25^+^Foxp3^+^ phenotype differing in terms of origin and mechanisms of action. The Foxp3 transcription factor is not sufficient for maintaining the Treg function and phenotype. It was demonstrated that Treg development, suppressive function, and plasticity potential are regulated by specific epigenetic changes (Ohkura et al. 2013). Regulatory B cells develop from various B cell progenitors and, similarly to Tregs, maintain self-tolerance and prevent autoimmunity. Various types of Bregs share similar suppressive mechanisms, and the most investigated are interleukin (IL)-10-producing regulatory B cells termed B10. Unlike CD4^+^CD25^+^Foxp3^+^ Tregs, which are defined by the expression of the Foxp3 transcription factor, there is no specific transcription factor identified for Bregs. However, interferon regulatory factor-4 was proposed as a candidate for the Breg identification marker (Matsumoto et al. 2014). Recently, Foxp3-expressing regulatory B cells were found in esophageal cancer and systemic lupus erythematosus patients (Shi et al. 2014; Vadasz et al. 2015), and additionally in collagen-induced arthritis DBA/1J mice (Park et al. 2016). Despite this latest discovery, it is not known if there is any specific “epigenetic signature” of different populations of regulatory B cells.

## Regulatory T Cells

Regulatory T cells (Tregs) play an important role in the immune system homeostasis. They are involved in the maintenance of immune tolerance to self-antigens and suppression of the excessive and harmful response to conventional foreign antigens. Tregs may prevent or inhibit the development of autoimmune diseases, allergies, and other types of hypersensitivities. They are involved in maintaining tolerance to fetal antigens and transplanted tissues as well as prevent graft-versus-host reaction. In contrast, an excessive suppressor activity of Tregs facilitates the development of cancer, inhibits immune response to various pathogens, and reduces the effectiveness of vaccines (Bluestone and Tang 2005; Wang 2006; Wing and Sakaguchi 2010). Various types of mouse and human T cells with suppressor activity have been described. They are distinguished based on the expression of specific markers and mechanisms of action (Table 1).

**Table 1:** Types of regulatory T cells

Regulatory T cell (mouse and human)	Function	Mechanism of suppression	References
tTreg and pTreg (CD4^+^CD25^+^Foxp3^+^)	Suppression of proliferation and cytokine secretion by CD4^+^CD25^−^ T cells Maintenance of homeostasis Protection against autoimmunity and hypersensitivity	Cell–cell contact IL-10 and TGF-β	Peterson (2012) Bettini and Vignali (2010)
Th3 (CD4^+^CD25^+^ variable expression of Foxp3)	Suppression of proliferation Th1 and Th2 cells Food tolerance	TGF-β	Gol-Ara et al. (2012) Apostolou et al. (2002)
Tr1 (CD4^+^CD25^−/low^)	Suppression of proliferation and cytokine secretion by naive CD4^+^CD25^−^ T cells, Th1, Th2	IL-10 and TGF-β	Zeng et al. (2015)
Treg (CD8^+^CD28^−^)	Suppression of the immune response by modulation of DCs Contribution to the development of transplantation tolerance	Interaction with ligand on DCs IL-10 and TGF-β	Gol-Ara et al. (2012) Churlaud et al. (2015)
Treg (CD8^+^CD28^+^)	Blockade of activation of naïve and effector T cells Suppression of IgG/IgE antibody synthesis IL-4 expression and proliferation of CD4^+^CD25^−^ T cells	Cell–cell contact IL-10, TNF-α, IFN-γ Granzyme B	Zhang et al. (2014) Churlaud et al. (2015)
γδ Treg	Suppression of anti-tumor and anti-infection responses	IL-10 and TGF-β	Kosten and Rustemeyer (2015) Ye et al. (2013)

*nTreg* natural regulatory T cell (recently named thymus-derived regulatory T cell, tTreg), *iTreg*-induced regulatory T cell (the recent name is peripherally derived Treg, pTreg)

Other types of regulatory T cells listed in the table are mainly peripherally induced regulatory T cells, which do not express Foxp3 or exhibit a low level of its expression. This table does not include all Tregs discussed in the literature but those detected in experimental models using transgenic mice or in vitro-induced Tregs

### Origin and Development of CD4^+^CD25^+^Foxp3^+^ Treg Cells

Treg cells of the CD4^+^CD25^+^Foxp3^+^ phenotype have been, to date, the most commonly studied type of regulatory T cells. They are crucial in the regulation of the immune response in both physiological and pathological conditions. Their history began in the 1990s when Sakaguchi et al. (1995) demonstrated that activated CD4^+^CD25^+^ T cells can maintain self-tolerance. Until recently, two types of CD4^+^CD25^+^Foxp3^+^ were distinguished: natural regulatory T cells (nTregs) and adaptive/induced regulatory T cells (iTregs) arising in peripheral lymphoid organs upon antigen activation of naïve CD4^+^CD25^−^ T cells (Curotto de Lafaille and Lafaille 2009; Bilate and Lafaille 2012). As presented in Table 1, different Treg subsets have been identified, but two major types expressing Foxp3 transcription factor can be distinguished based on their origin: thymus-derived Tregs (tTregs), until recently commonly called “natural regulatory T cells”; and peripherally derived induced Tregs (pTregs), formerly known as “induced Tregs” (Elkord 2014). According to the new terminology, thymus-derived regulatory T cells are the major mediators of central immune tolerance, whereas peripherally derived regulatory T cells are involved in the regulation of peripheral immune tolerance in sites of inflammation, in oral, mucosal, and fetal tolerance (Yadav et al. 2013). In addition, in vitro-induced/in vitro-derived regulatory T cells are distinguished based on the potential to stably express Foxp3 (Abbas et al. 2013). Moreover, there are other regulatory T cells found in the periphery, which did not express Foxp3 (Tr1) or demonstrate a variable level of its expression (Th3) (Gol-Ara et al. 2012; Zeng et al. 2015). The role and origin of non-CD4^+^ regulatory T cells such as CD8^+^ Tregs or Tγδ regulatory cells still remain unclear (Cone et al. 2008; Fontenot et al. 2003; Xystrakis et al. 2004; Ye et al. 2013).

**Fig. 1:**
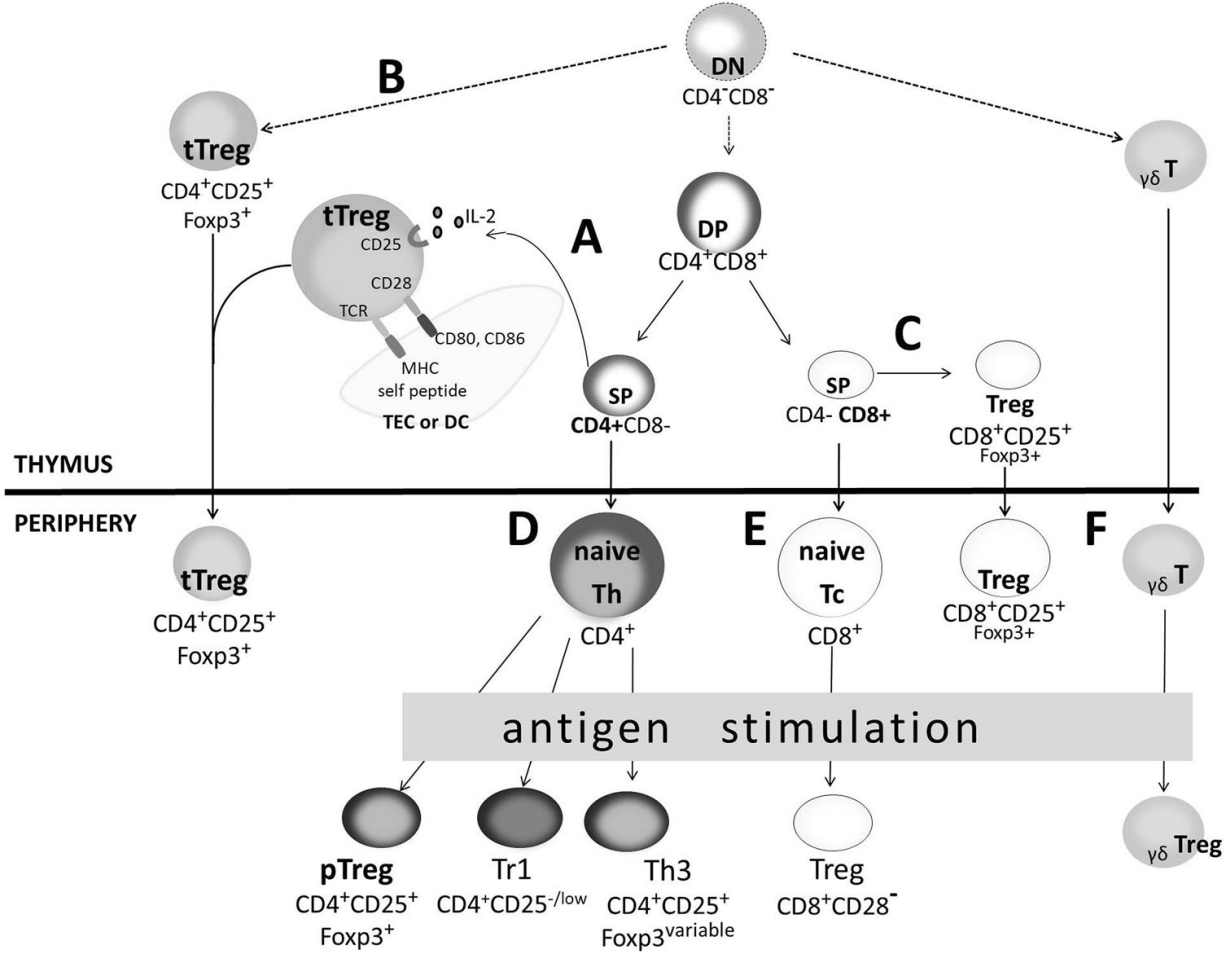
Regulatory T cell development (Churlaud et al. 2015; Josefowicz and Rudensky 2009; Kosten and Rustemeyer 2015; Zhang et al. 2014). Thymus-derived regulatory T cells (tTregs) can develop from SP CD4^+^CD8^−^ thymocytes (**a**), or an alternative pathway of their development from DN is hypothesized (**b**); CD8^+^CD25^+^Foxp3^+^ Tregs can also arise in the thymus from SP CD8^+^CD4^−^ thymocytes (**c**). Both thymic Tregs migrate to peripheral lymphoid organs as mature T cells exhibiting suppressive potential. Peripherally induced Tregs differentiate from antigen-activated naive Th CD4^+^ cells into pTregs of CD4^+^CD25^+^Foxp3^+^ phenotype. Additionally, Tr1 and Th3 are generated (**d**); naive T CD8^+^ cells can differentiate into CD8^+^CD28^−^ Tregs (**e**); γδ Tregs can arise from antigen-activated γδ T cells (**f**). *DN* double-negative, *DP* double-positive, *SP* single-positive, *TEC* thymic epithelial cells, *DC* dendritic cells

The origin and development of both thymus-derived and peripherally induced regulatory T cells are still studied. Itoh et al. (1999) showed that tTregs of the CD4^+^CD25^+^Foxp3^+^ phenotype may occur at a late stage of single-positive (SP) CD4^+^ thymocyte development (Fig. 1a). According to the “strict-affinity model,” it is assumed that tTregs derive from the thymocyte lineage characterized by a high affinity of T cell receptor (TCR) to the MHC II/self-peptide complexes. Signal transduction from the high-affinity TCR/CD3 complex determines the start of the transcription program, which is responsible for the differentiation of immature thymocyte into mature tTreg (Liston and Rudensky 2007). The CD3 ζ chain phosphorylation level can be used as a marker of signal strength transmitted from the TCR/CD3 complex, and it was highly increased in CD4^+^CD25^+^ thymocytes as compared to CD4^+^CD25^−^ (Shevach 2006). Thymocyte selection targeted towards tTreg development may be explained by the “limiting niche hypothesis.” The development of thymus-derived Tregs requires two signals: one derived from the high affinity TCRs and the other delivered by limited access factors such as IL-2 or CD28 ligands (CD80/CD86). Conversion of thymocytes with intermediate TCR affinity into tTregs is not restricted by IL-2 and CD80/CD86 molecules. In contrast, conversion of thymocytes with high TCR affinity into tTregs requires IL-2 and CD80/CD86 molecules. The interaction between CD28 and CD80/CD86 molecules promotes tTreg survival possibly by affecting the production of IL-2 and induction of anti-apoptotic protein Bcl-2. Co-stimulation through the CD28 molecule is essential for thymus-derived Tregs development and maintains their stable pool in the peripheral lymphoid organs. It is not known if these signals must be provided at the same time, however, they are necessary to induce the expression of the Foxp3 transcription factor (Liston and Rudensky 2007). The results of recent studies suggest an additional pathway of tTreg development starting from the stage of double-negative (DN) CD4^−^CD8^−^ thymocytes when the rearrangement of TCR encoding genes is not completed (Fig. 1b) (Pennington et al. 2006). This suggests that tTreg selection may be at least partially independent of the TCR signal (Hsieh et al. 2012). There is relatively little direct data to argue that the repertoire of tTregs differs from that of conventional CD4 T cells. There are some experimental results and theoretical considerations in this matter. In the thymus, T cells with high affinity TCRs for self-peptide/MHC complexes undergo programmed cell death, depriving the conventional T cell pool of the majority of autoreactive cells (Starr et al. 2003). Thymic regulatory T cells are generated in response to high-affinity interactions with self-peptide/MHC complexes (Lio and Hsieh 2008). Thus, it is largely considered that tTregs cells have a high level of self-reactivity compared to conventional CD4 T cells. This led to the hypothesis that thymus-derived Tregs mediate their function in the peripheral lymphoid organs by responding to self-peptide/MHC II complexes, but not to foreign ones. This assumption was verified by the results demonstrating the usage of a different range of TCR α chains by Tregs and conventional CD4 T cells (Hsieh et al. 2004). The repertoire of Tregs is highly diverse and is overlapping, to some extent, with the repertoire of conventional CD4 T cells (Hsieh et al. 2006; Pacholczyk et al. 2006; Pacholczyk and Kern 2008).

It was also demonstrated that Foxp3 expression might occur at the DN stage of thymocyte development. The highest percentage of Foxp3^+^ thymocytes was detected in the SP CD4^+^ thymocyte subset, and gradually decreases in double-positive CD4^+^CD8^+^, SP CD8^+^, and DN thymocytes (Fontenot et al. 2005). Similarly, Foxp3 expression was found in human DN thymocytes (Tuovinen et al. 2008). It is commonly considered that thymic regulatory T cells follow the conventional T cell developmental stages determined by the expression of CD4 and CD8 markers. A two-step model of tTreg differentiation is widely accepted and is based on the assumption that TCR/CD28 signals induce the generation of tTreg precursors from immature SP CD4^+^ thymocytes. In physiological conditions, the conversion of self-reactive SP CD4^+^ thymocytes into tTregs requires positive selection involving thymic cortical epithelial cells with high expression of MHC II/self-peptide complexes. Next, thymic dendritic cells (DCs) are necessary to deliver costimulatory signals in the presence of IL-2 and possibly other γ-chain cytokines or other less-known factors. In such conditions, immature tTregs characterized by the CD4^+^CD25^+^ phenotype are converted to mature CD4^+^CD25^+^Foxp3^+^ thymus-derived regulatory T cells (Lio and Hsieh 2008). This hypothetical two-step model was documented also by in vitro studies in polyclonally pre-activated thymocytes co-cultured with JAWS II cells delivering costimulatory signals (Bienkowska et al. 2014). Foxp3 is a key lineage-defining transcription factor important for the development and suppressive function for tTregs in mice (Fontenot et al. 2003; Hori et al. 2003) and humans (Roncador et al. 2005).

### Origin and Development of Other Treg Cells

Other types of Treg cells such as CD8^+^CD25^+^ are also developed in the thymus (Fig. 1c) and express several molecules characteristic of tTregs, namely, CD25, Foxp3, CTLA-4, and glucocorticoid-induced tumor necrosis factor (TNF) receptor (GITR). Similarly to tTregs, the suppressive mechanism exerted by this population is cell contact-dependent; hence, they are also called natural or thymic CD8^+^ Tregs. CD8^+^CD28^+^ Tregs inhibit priming of CD8^+^ and CD4^+^ T cells, and antibody-mediated response against oral antigens (Table 1). The γδ T cells are commonly of the CD8^+^Foxp3^−^ phenotype and are found in the periphery, mainly in the intestinal epithelium (Fig. 1f). They are primarily suppressive and are associated with mucosal tolerance, but can also regulate autoimmunity and tumor immunity by producing IL-10 and transforming growth factor (TGF)-β similarly to Tr1 cells (Kosten and Rustemeyer 2015). Moreover, CD8^+^CD28^−^ Tregs (Fig. 1e) can be induced in the periphery from naïve CD8^+^ T cells upon activation by allogenic antigen-presenting cells (APCs) or monocytes, in the presence of IL-2 and granulocyte macrophage-colony stimulating factor (GM-CSF). This population is observed in tonsils, but rarely detected in peripheral blood (Gol-Ara et al. 2012; Zhang et al. 2014).

Various types of regulatory T cells are induced upon antigen stimulation in peripheral lymphoid organs. Naive CD4^+^ T helper (Th) cells can differentiate into CD4^+^CD25^+^Foxp3^+^ pTregs, Th3, and Tr1 (Fig. 1d). Peripherally induced CD4^+^CD25^+^Foxp3^+^ Tregs can arise under low-dose antigenic stimulation or in a particular cytokine environment (TGF-β, IL-10, and IL-2). The mechanism by which TGF-β induces transcription of Foxp3 involves cooperation of Smad2/3 and nuclear factor of activated T cells (NFAT) (Chen et al. 2003; Tone et al. 2008) and STAT3/5 at a *foxp3* gene enhancer element (in the promotor and CNS2 region, respectively) (Burchill et al. 2007; Zheng et al. 2007), whereas IL-2 activates the STAT5 transcription factor, which binds the *foxp3* gene and co-acts with STAT3, which results in the induction of Foxp3 expression. IL-2 is required for TGF-β-induced Foxp3 transcription in vitro and suppressive activity of Tregs (Zheng et al. 2004; Zorn et al. 2006). It may replace the requirement for CD28 co-stimulation for the induction of Foxp3 by anti-CD3 monoclonal antibodies and TGF-β (Zheng et al. 2007). Although it is known that both tTregs and pTregs express Foxp3, its role in the development and function of other induced Treg cells, is still not fully explained. Although some researchers detected Foxp3 expression in Th3 cells, it is rather considered that its expression is variable. Th3 cells can be induced from naive CD4^+^ T cells by TGF-β, and have a significant role in oral tolerance to foreign antigens and contribute to the suppression of autoimmune response. Tr1 can be generated in vitro by continuing TCR stimulation in the presence of high levels of IL-10 and IL-15. It is also possible that interferon (IFN)-α is critical for efficient differentiation of Tr1 in addition to IL-10 in vitro. Moreover, molecules such as CD2 and CD46 can induce Tr1 differentiation by interacting with CD58. The major mechanisms of suppression by Tr1 cells are based on contact-independent pathways, particularly via cytokines IL-10 and TGF-β, that can also act in contact-dependent mechanisms (Bettini and Vignali 2010; Peterson 2012; Zeng et al. 2015). Moreover, CD8^+^CD28^−^ Tregs (Fig. 1e) can be induced in the periphery from naïve CD8^+^ T cells upon activation by allogeneic APCs or monocytes, in the presence of IL-2 and GM-CSF. This population is observed in tonsils, but is rarely detected in peripheral blood (Gol-Ara et al. 2012; Zhang et al. 2014). The γδ T cells are commonly of the CD8^+^Foxp3^−^ phenotype and are found in the periphery, mainly in the intestinal epithelium (Fig. 1f). They are primarily suppressive and are associated with mucosal tolerance, but can also regulate autoimmunity and tumor immunity by producing IL-10 and TGF-β similarly to Tr1 cells (Kosten and Rustemeyer 2015).

### Suppressive Mechanisms of Thymus and Peripherally Derived CD4^+^CD25^+^Foxp3^+^ Treg Cells

Regulatory T cells are able to suppress a wide range of immune cells including CD4^+^ and CD8^+^ T cells, natural killer (NK) cells, B cells, NK T cells, monocytes, and DCs. The primary and best characterized function of Tregs is the inhibition of proliferation and cytokine production by effector-activated CD4^+^CD25^+^ T cells.

**Fig. 2:**
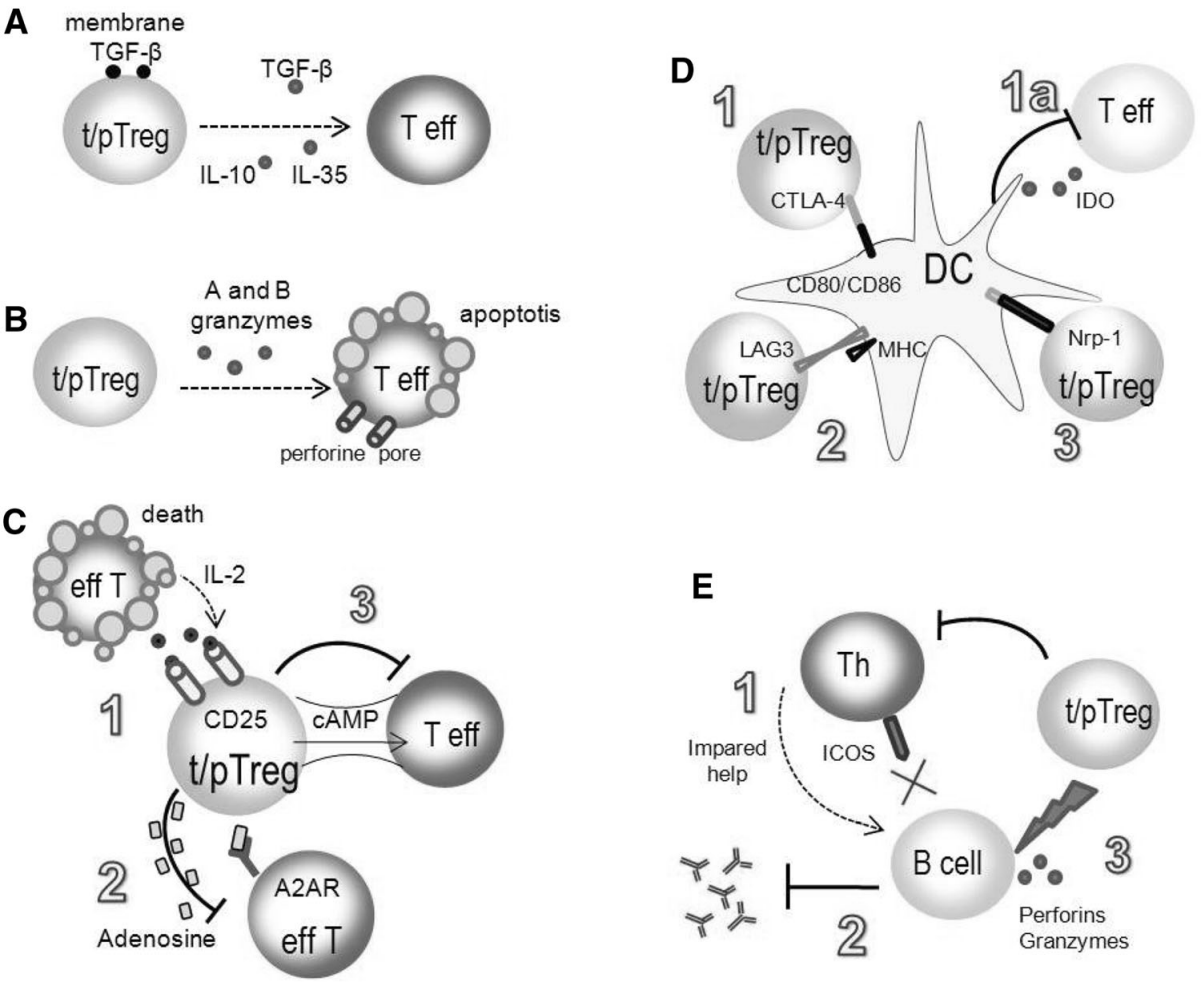
Mechanisms of regulatory T cell suppression (Schmidt et al. 2012; Shevach 2009; Vignali et al. 2008). Cytokines IL-10, IL-35, and TGF-β can mediate the suppressive activity of tTregs and pTregs by inhibiting cytokine production and proliferation of effector T cells (**a**); Tregs can exhibit the cytotoxic activity by the mechanisms involving perforin and granzymes release (**b**); effector T cells can be inhibited through metabolic disruption: high expression of CD25 molecules on Treg cells leads to efficient binding of IL-2 and to apoptosis of Teff cells (**c**.*1*); CD39 and CD73 ectoenzymes presented on Tregs participate in the formation of extracellular adenosine which binds A_2A_R on the effector T cells leading to their suppression (**c**.*2*); inhibition of effector T cells can be mediated by cAMP (**c**.*3*); Tregs can inhibit the activity of dendritic cells by CTLA-4 and CD80/CD86 ligation, which weakens the costimulatory signal delivered to effector T cell (**d**.*1*). The interaction of CTLA-4 with CD80/CD86 induces IDO that catalyzes the conversion of tryptophan to kynurenine and other pro-apoptotic metabolites (**d**.*1a*); LAG-3/MHC II interaction induces the suppression of dendritic cells (**d**.*2*) Neuropilin-1 prolongs the interaction between Tregs and dendritic cells, resulting in limited access of effector T cells to dendritic cells (**d**.*3*); Tregs inhibit B cell maturation by blocking T-helper cells through the downregulation of inducible T cell costimulator (ICOS; **e**.*1*); Tregs can inhibit the synthesis of antibodies and Ig class switching (**e**.*2*); Tregs can kill B cells by perforin and granzyme B release (**e**.*3*)

The suppressive mechanisms of regulatory T cells are the subject of intense studies. The mechanisms described so far include synthesis of inhibitory cytokines, cytolysis, metabolic disruption, modulation of DC maturation, or the function and suppression of B cells (Fig. 2).

#### Production of Inhibitory Cytokines

Tregs produce IL-10, IL-35, and TGF-β, which mediate the suppression of target cells (Fig. 2a). IL-10 inhibits cytokine production by effector CD4^+^ and CD8^+^ T cells and activated macrophages, and reduces the expression of MHC II and ICAM-1, CD80, CD86 molecules on APCs. The role of IL-10 in the suppressive mechanism of Tregs is widely documented (Chaudhry et al. 2011; Sojka and Fowell 2011). Mice unable to produce or bind IL-10 by membrane receptors are more vulnerable to acute graft rejection due to the inefficient activity of Treg cells. The contribution of TGF-β has not been thoroughly proven. TGF-β, as opposed to IL-10, can exist in soluble and membrane forms. It is postulated that the membrane TGF-β may be implicated in the delivery of inhibitory signal to the target cell during the direct contact with Treg. The suppressive activity of TGF-β is directed against activated macrophages, T and B lymphocytes, and NK cells. TGF-β, similarly to IL-10, inhibits the expression of MHC II on APCs. In the presence of high concentrations of IL-2, TGF-β induces an increase in the expression of CD25, CD122, and CTLA-4 molecules on naïve CD4^+^ T cells, resulting in their activation and differentiation into peripherally induced Treg cells (Zheng et al. 2004).

Recently discovered IL-35 belongs to IL-12 cytokine family. This cytokine is secreted only by Tregs and was found to contribute to their suppressive function (Collison et al. 2007). It was demonstrated that ectopic expression of IL-35 activates regulatory functions in naïve T cells and secreted IL-35 inhibits the proliferation of effector T cells in vitro. It is not known whether IL-35 inhibits the development and function of other immune cells such as DCs or macrophages. Tregs producing IL-35 are derived from peripherally induced naïve Th cells; however, their suppressive activity did not require the transcription factor Foxp3. The biological activity of this newly discovered cytokine requires further studies (Olson et al. 2013).

#### Target Cell Cytolysis

So far, the cytotoxic function was largely attributed to NK and cytotoxic CD8^+^ T cells (Lieberman 2003). However, it has been found that human and murine regulatory T cells can also exhibit a cytotoxic activity (Fig. 2b). Human Tregs induce the apoptosis of target effector T cells by perforin and granzyme A. Murine Tregs use granzyme B in a perforin-dependent or independent manner to induce the apoptosis of target T cells. Recently, it was discovered that TRAIL-DR5 (TNF-related apoptosis-inducing ligand-death receptor 5) and galectin-1 may participate in the induction of apoptosis in T cells (Beeston et al. 2010; Choi et al. 2013).

#### Metabolic Disruption

This mechanism is based on the competition of Treg and effector T cells for IL-2 (Fig. 2c.1). High expression of IL-2 receptor α chain (CD25) on Tregs allows for an efficient binding of IL-2, which is important in maintaining Treg homeostasis in vivo (Yu et al. 2009) and suppressive activity in vitro (Thornton et al. 2004). According to the results of other studies, Tregs can suppress effector T cells by inactivating the transcription of *IL*-*2* mRNA (Oberle et al. 2007). Results of the studies performed in the hybrid system have shown that human Treg cells are able to inhibit the proliferation of effector T cells, but the addition of the anti-CD25 blocking antibody had no effect on the suppressive function of human Tregs, leaving the role of IL-2 questionable (Tran et al. 2009). An additional suppressive mechanism relies on the induction of intra- and extracellular secretion of adenosine nucleotides. Expression of CD39 and CD73 ectoenzymes is responsible for the formation of extracellular adenosine, which is bound by A_2A_R (adenosine receptor 2A) on effector T cells, resulting in the inhibition of their effector function. The binding of the adenosine by A_2A_R inhibits the expression of IL-6, which takes part in the development of pro-inflammatory Th17 cells and TGF-β synthesis inducing Foxp3 expression and Treg development (Fig. 2c.2). Another mechanism of effector T cell suppression can be mediated by the cyclic AMP (cAMP), which inhibits the proliferation and IL-2 synthesis by conventional CD4 T cells (Bopp et al. 2007). It was shown that the expression of cAMP was high in Tregs in contrast to conventional CD4^+^ T cells. Direct transfer of a strong inhibitor cAMP through the gap junctions, observed in co-cultures of effector T and Treg cells, causes an increase of cAMP level in CD4^+^ T cells, suggesting the participation of cAMP in Treg suppressive activity (Fig. 2c.3) (Borsellino et al. 2007; Deaglio et al. 2007).

#### Modulation of DC Function

Regulatory T cells can modulate the maturation and antigen-presenting function of DCs necessary for the activation of effector T cells. CTLA-4, which is constitutively expressed on Tregs, is involved in this process (Takahashi et al. 1998). Deletion of *ctla*-*4* results in the development of autoimmune diseases (Wing et al. 2008). CTLA-4 can be involved in the suppressive activity of Treg cells in two different ways: (1) interaction of CTLA-4 with CD80/CD86 costimulatory molecules on DCs results in the inhibition or reduction of their expression. The decrease of CD80/CD86 expression on APCs weakens the costimulatory signal delivered to effector T cell leading to suppression of their activation (Fig. 2d.1) (Onishi et al. 2008); (2) interaction of CTLA-4 with CD80/CD86 molecules on APCs induces the indoleamine 2,3-dioxygenase (IDO), which catalyzes the conversion of tryptophan to kynurenine and other pro-apoptotic metabolites, leading to inhibition of the activation of effector T cells (Fig. 2d.1a) (Baban et al. 2009).

LAG-3, a CD4 homologue that binds MHC II with very high affinity, is a surface molecule involved in the suppression of DCs (Fig. 2d.2). LAG-3/MHC II interaction induces the intracellular inhibitory signaling pathway through the immunoreceptor tyrosine-based activation motif sequence, whereby it inhibits the maturation of DCs and their ability to activate naïve T cells. Extracellular signal-regulated kinase, SHP-1 phosphatase, and FcγRγ receptors were found to participate in this pathway (Liang et al. 2008). Treg suppressor activity may also be mediated by neuropilin-1 (Fig. 2d.3), which prolongs the interaction between the Treg and DC by restriction of the access of the effector T cell to DC and lowering effectiveness of antigen presentation (Delgoffe et al. 2012).

#### Direct and Indirect Suppression of B Cells

Currently, interactions between Tregs and B cells are largely investigated, especially due to the role of B cells in the pathogenesis of autoimmune diseases. Tregs can modulate B cells’ response indirectly by impairing their maturation, blocking Th cells through the downregulation of inducible T cell costimulator (Fig. 2e.1), or directly by inhibition of antibody production and immunoglobulin class switching in the absence of Th cells (Fig. 2e.2). Moreover, regulatory T cells can kill B cells mainly by releasing perforin and granzyme B (Fig. 2e.3). In conclusion, Tregs play an important role in the regulation of B cells immune response especially by preventing autoantibodies synthesis (Mahnke et al. 2008; Wang and Zheng 2013).

The day/night variation of Treg development and suppressive activity is an interesting aspect of their biology and function and can be regulated by glucocorticoids (GC), through the glucocorticoid receptors (GCR) of Tregs. Kiernozek et al. (2014) have shown, in a mouse model, that during sleep (during the day), when the body is at rest, the percentage of thymus-derived Tregs and their suppressive activity as well as GCR expression and plasma concentrations of GC were decreased. During the active phase (at night for a mouse), an increase in the percentage and suppressive activity of Tregs, as well as in the expression of GCR and concentration of GC were observed. Furthermore, gender-dependent differences were demonstrated for tTreg development, suppressive activity, and GCR expression. The percentage of tTregs and their suppressive activity were higher in male mice (Kiernozek et al. 2014). These results thus far support the documented sex-dependent dimorphism of the immune system (Libert et al. 2010; Moldovan et al. 2008; Pelfrey et al. 2002). Taken together, the day/night rhythmicity of tTreg development and activity and sex-dependent differences are important components of the immune response, frequently neglected in clinical procedures concerning, for example, vaccination or immunosuppressive therapy.

### Origin and Development of CD4^+^CD25^+^Foxp3^+^ Treg Cells as a Result of Epigenetic Modifications

Foxp3 protein is widely considered a specific marker for Treg cells. However, the concept of Foxp3 as a “lineage-specifying factor” of Tregs is an oversimplification, especially in humans (Fontenot et al. 2003; Hori et al. 2003; Roncador et al. 2005). Molecular biology experiments revealed that the development and function of stable Treg cell is governed not only by Foxp3 activity, but also by epigenetic mechanisms (Arvey et al. 2015; Floess et al. 2007; Kanno et al. 2012). These mechanisms, including DNA methylation, histone modifications, nucleosome positioning, as well as microRNAs expression, are responsible for the regulation of gene expression in hematopoiesis and development and activity of immune cells (Huehn and Beyer 2015; Suarez-Alvarez et al. 2012). In particular, methylated DNA sequences are “silenced”, while demethylation is linked to opening of the transcription sites and increased gene expression. Chromatin remodeling plays a role in determining the accessibility of genes by transcriptional activators or repressors. Very interesting are results obtained by Samstein et al. (2012) that suggest a model for control of Treg cell differentiation and function by Foxp3 through a network of preformed enhancers and co-factors operating yet in precursor cells.

Differentiation of T cells, their response to “danger” signals, and the induction of immune tolerance are regulated by dynamic epigenetic modifications (Cuddapah et al. 2010; Wei et al. 2009). Treg development is also governed by characteristic epigenetic modifications (Kitagawa et al. 2013; Li et al. 2014). Recently, it has been shown that allergies, tumors, and many autoimmune diseases, such as rheumatoid arthritis and systemic lupus erythematosus (SLE), are caused by deregulation of epigenetic mechanisms in Treg cells (Cuddapah et al. 2010; Kanno et al. 2012).

In the thymus, after TCR stimulation, thymocytes undergo independent, but complementary, changes leading to Treg maturation (Ohkura et al. 2012, 2013). Epigenetic mechanisms change expression of the following genes: *Foxp3*, *Tnfrsf18* (encoding GITR), *Ctla4*, *Il2ra* (encoding CD25), and *Ikzf4* (encoding Eos) (Delgoffe et al. 2012; Marson et al. 2007; Ohkura et al. 2012). Normal and stable expression of these genes ensures development of Tregs and controls their suppressive functions. In parallel, the cells start to express Foxp3. As a consequence, tTregs become functionally stable in peripheral lymphatic organs, whereas in pTregs, the so-called physiological instability is observed. Some authors suggest a new acronym “tTregMe^+^Foxp3^+^” (thymic Treg cells, epigenetic pattern+, Foxp3 protein+) describing functionally and phenotypically stable Tregs.

The *forkhead box P3* gene (*Foxp3*) encoding the Foxp3 transcriptional regulatory protein is believed to be the master regulator gene for Tregs (Hori et al. 2003). *Foxp3* gene expression is controlled by four elements, containing conserved, non-coding sequences (CNS): The promoter region, CNS1, CNS2 (localized in the first intron), and CNS3 (in the second intron) (Mantel et al. 2006). These sites are regulated by epigenetic modifications characteristic of Tregs that determine the chromatin structure and DNA methylation, thus altering the accessibility of the gene locus to transcription factors (Huehn and Beyer 2015; Passerini et al. 2014).

#### Foxp3 Promoter

In this region, CpG motifs are entirely demethylated in tTregs, but partially methylated in conventional Th cells (Floess et al. 2007). In the case of pTreg, the methylation pattern initially remains identical to the conventional Th cells (Mantel et al. 2006; Polansky et al. 2008). This may explain the instability of function of pTreg and plasticity of pTreg and conventional Th cells (Kitagawa et al. 2013; Ohkura et al. 2013; Schmidl et al. 2009; Wei et al. 2009). However, as a result of repetitive TCR stimulation in the presence of TGF-β and IL-2, decreased methylation of CpG islands in the *Foxp3* promoter of pTreg was observed, as low as to the level characteristic for tTreg (Burchill et al. 2007; Huehn and Beyer 2015; Kim and Leonard 2007).

DNA methyltransferase and heterochromatin protein 1 act as a repressor of *Foxp3* promoter in SP CD4^+^ thymocytes and conventional Th cells. DNA methylation and histones acetylation/methylation result in limited access of transcription factors to the DNA and suppress the expression of *Foxp3* (Janson et al. 2008). This state of the promoter might be changed by an appropriate TCR stimulation through auto- and alloantigens (in the thymus and peripheral lymphoid tissues, respectively) and a particular cytokine environment (Overacre and Vignali 2016). After TCR stimulation, PIAS1 protein becomes deleted and, consequently, demethylation of the promoter and reduced methylation of histone H3 occurs. *Foxp3* promoter becomes available for transcription factors which induce gene expression (Liu et al. 2010). From the point of view of researchers, epigenetic modification can be done using DNA demethylation and histone protein acetylation of the Foxp3 gene locus. As a consequence, Foxp3 expression could be induced in naïve T cells that may lead to the differentiation of these cells to Tregs (Moon et al. 2009).

#### CNS1 (TGF-β Sensitive)

CNS1, an “enhancer” region in the *Foxp3* locus, contains binding sites for NFAT and Smad3 (Schlenner et al. 2012). Some investigators have shown that Foxp3 forms protein complexes with NFAT and displaces AP-1 in the NFAT complex in its DNA-bound form (Wu et al. 2006), which suggests that Foxp3 might replace AP-1 at activation-dependent enhancers in Treg cells (Samstein et al. 2012; Wu et al. 2006). CNS1 is regulated only by histone modifications (acetylation/methylation), which limit or provide access to DNA. This region in both tTregs and induced Tregs is characterized by histone acetylation and complete DNA demethylation. The CNS1 region is sensitive to stimulation by TGF-β, which activates histone deacetylase (HDAC). This is true only for Tregs that are induced from a conventional Th cell in the peripheral lymphoid organs and in vitro (Passerini et al. 2014). In CNS1 knockout animals, normal development of tTregs was observed with a simultaneous lack of the induction of pTreg cells at the periphery (Zheng et al. 2010).

CNS3 (pioneer element) is called a pioneer because of its role in the initiation of Foxp3 transcription. The chromatin structure is open at this site and is easily accessible for the transcription factors. Chromatin modifications at this site show permissive marks (H3K9/14Ac, H3K4me2 and H3K4me1) in Tregs, but also mono- (H3K4me1) and di- (H3K4me2) methylation in Treg-precursors (CD4^+^CD8^+^ and CD4^+^CD8^−^ thymocytes) (Wei et al. 2009). Cooperation of CNS3 with transcription factors (the members of the NF-κB family) results in open Foxp3 locus in the regions of CNS1 and CNS2. Interestingly, the activity of CNS3 is not necessary when the Foxp3 is expressed (Huehn and Beyer 2015). CNS3^−/−^ mice have significantly reduced Treg numbers, but normal per cell levels of Foxp3 occur in the remaining Tregs (Zheng et al. 2010). This may explain the observation that extracellular Foxp3 was bound mainly to enhancers already accessible in precursor CD4^+^Foxp3^−^ T cells prior to Foxp3 expression with only 2% of all Foxp3-bound enhancers observed in Foxp3^+^ Treg cells, but not in resting Foxp3-negative T cells (Samstein et al. 2012).

CNS2 (Treg-specific demethylated region, also called TSDR) is a highly conserved, CpG dinucleotide-rich region in both mouse and human Th cells. It is completely demethylated in tTreg, but methylated in conventional Th cells. Importantly, the epigenetic modifications enhancing transcriptional activity and determining stable protein expression of Foxp3 (Samstein et al. 2012) are found in the TSDR region (Kim and Leonard 2007; Zheng et al. 2010).

Maintenance of TSDR demethylation is critical for stable Foxp3 expression and maintenance of the Tregs suppressive function. tTregs are stably demethylated at the TSDR, even after they leave the thymus. pTregs exhibit low levels of TSDR methylation, which can be additionally reduced after a strong TCR activation in the presence of TGF-β and IL-2 (Polansky et al. 2008). After TCR activation, NFAT is responsible for constitutive expression of Foxp3 in proliferating Tregs. NFAT binding to CNS2 leads to conformational changes in the DNA structure. CNS2 get closer to Foxp3 promoter (Tone et al. 2008). It seems that such a system ensures stability of new pTregs. Interestingly, TSDR demethylation is not necessary for initiation but is indispensable for constitutive and long-term Foxp3 expression. This region contains multiple transcription factor binding sites (Marson et al. 2007; Polansky et al. 2010). Epigenetic modifications of TSDR region in Th cells allow for fast and necessary mobilization of the immune system, depending on the surrounding environment. For example, the presence of TGF-β, IL-2, IFN-γ induces demethylation of TSDR and development of pTregFoxp3^+^ (Chen et al. 2011; Daniel et al. 2015). On the other hand, Toll-like receptor (TLR) and/or IL-12, IL-4, IL-6 signal determines methylation of TSDR, inhibition of Foxp3 expression, and conversion into Th2, Th1, or Th17 (Suarez-Alvarez et al. 2012). Epigenetic mechanisms are crucial for homeostasis; however, they are also involved in pathologic activity of Tregs in autoimmune diseases, chronic infections, or tumors. It was shown that in the state of homeostasis (at a steady state), stable expression of Foxp3 is possible even after deletion of TSDR (Li et al. 2014). In inflammatory settings, functional TSDR supported high activity of Tregs (Zorn et al. 2006). Exposure of Treg to pro-inflammatory cytokines, including IL-4, IL-6, low levels of IL-2, or strong TCR-activating signal results in the instability of Tregs.

TSDR is highly methylated in other populations of immune cells, which results in complete deactivation of the Foxp3 gene. This state might be rescued using an epigenetic modifier, e.g., DNA methyltransferase inhibitor—5AzaD or HDAC inhibitor—trichostatin A (Anderson et al. 2014). These factors induce Foxp3 expression in cytotoxic T cells or NK cells (Josefowicz and Rudensky 2009; Zorn et al. 2006). Treatment of naïve CD4^+^CD25^−^Foxp3^−^ T cells with DNA methyltransferase inhibitor or HDAC inhibitor induces differentiation into CD4^+^CD25^+^Foxp3^+^ Treg cells. Moreover, Treg cells induced by epigenetic modification showed regulatory activity on allogeneic Th cells (Moon et al. 2009).

Autoimmune diseases and chronic infections are strongly related to abnormal activity of the immune system. The main reason for this dysfunction is a lack of regulation of percentage and activity of Tregs (Anderson et al. 2014). It seems possible that analysis of the TSDR region methylation pattern may be helpful in diagnostics of Treg activity in patients and monitoring of Treg activity during the therapy (Lu et al. 2013). Ngalamika et al. (2015) identified differences in methylation of TSDR in patients with active SLE—very high methylation, or with psoriasis—high methylation. On the other hand, TSDR methylation below the level observed for control Tregs was observed in patients with bacterial and fungal infections (Ngalamika et al. 2015). Liu and Li (2015) observed that TSDR was demethylated, and Foxp3 was upregulated in patients with chronic viral infections (hepatitis B and C virus) and these changes were correlated with the development of hepatocellular carcinoma.

Considering the plasticity of pTregs, the assessment of methylation in *Tnfrsf18* (GITR), *CTLA4*, *IKZF2* (HELIOS), *LRRC32* (GARP, Treg activation marker), and *IL2ra, IL7R* should be assessed, as these factors are important for the development of “pTregMe^+^Foxp3^+^”. A library of epigenetic patterns in Tregs in various diseases might become a useful diagnostic tool. Dominguez-Villar et al. (2011) did not observe changes in TSDR methylation in cells obtained from patients with active multiple sclerosis in comparison to normal donors. The authors showed, however, that IL-12-dependent suppressive functions are weaker in this group. Tregs exerted Th1-like functions (IFN-γ production), changed the methylation profile of *CTLA4*, *IL10, TGF*-*β,* and *CCR5* and downregulation of these genes (Dominguez-Villar et al. 2011).

Bending et al. (2014) observed the lack of constitutive expression of Foxp3 in Tregs, despite high demethylation of the Foxp3 promoter and the TSDR region in chronic inflammation settings in patients with juvenile idiopathic arthritis. These cells were characterized by an abnormal IL-2 signaling pathway. Moreover, Foxp3 was not deubiquitinated and remained unstable (Bending et al. 2014).

Interestingly, analysis of the epigenetic pattern of Tregs might be considered a reliable diagnostic method. Demethylation of CpG in CNS1 of Foxp3 is typical of Tregs and might be used to identify Tregs in patients. Methylation analysis in circulating Tregs in patients with acute coronary syndrome revealed a low number of Tregs in comparison to the healthy population. The difference was not observed in flow cytometry analysis, which suggests a higher sensitivity of methylation analysis (Lu et al. 2013).

Epigenetic mechanisms stabilize the activity of Foxp3 and Treg cells function. New tools for regulation of epigenetic mechanisms may result in the development of new, effective therapeutic strategies. Currently, two groups of “epigenetic drugs” are available: DNA methyltransferase (DNMT) inhibitors and HDAC inhibitors (Altucci and Rots 2016; Lopez-Pastrana et al. 2015). DNMT inhibitors strongly induce expression of Foxp3; however, their use is limited because of high toxicity and induction of Th1 and Th2 cytokines. HAT/HDAC plays an important role in the stabilization of the Foxp3 function. Immunomodulatory activities of HDAC inhibitors were shown in various experimental models of inflammation, autoimmune diseases, and transplantation (Lopez-Pastrana et al. 2015; Wang et al. 2009).

## Regulatory B Cells

It is commonly considered that B cells can transform into antibody-secreting cells that play an important role in the immune response against pathogens and maintenance of immune homeostasis. However, they also release a variety of cytokines, play the role of APCs, and finally, contribute to the maintenance of tolerance (Harris et al. 2000; Mauri 2010; Shlomchik et al. 2001). The involvement of B cells in the regulation of cellular and humoral immune responses to pathogens or self-antigens is still not widely accepted despite a series of confirming experiments (Bergmann et al. 2001; Constant et al. 1995; Shen et al. 2003, 2014). In addition, it was demonstrated that mice lacking B cells develop a variety of defects in lymphoid organogenesis (Golovkina et al. 1999; Shen et al. 2003). The most intriguing abnormalities concern the decrease in T cell numbers in the thymus and spleen or a loss of follicular and splenic DCs (Fu et al. 1998; Joao et al. 2004; Kabashima et al. 2005; Ngo et al. 2001). B cells can regulate T cell responses by playing the role of APCs especially when antigen load is low (Bouaziz et al. 2007; Townsend and Goodnow 1998). They can produce cytokines modulating CD4^+^ T cell functions when stimulated by antigens or TLR ligands (Lund 2008) and promote the conversion of CD4^+^ T cells into Foxp3^+^ regulatory cells by producing TGF-β (Shah and Qiao 2008).

The hypothesis that B cells can contribute to the suppression of immunity dates back to 1974 when their role in suppressing delayed-type hypersensitivity (DTH) response was demonstrated (Katz et al. 1974; Neta and Salvin 1974). Very early attempts to identify Breg cells came from observations that B cell-lacking mice suffered a very severe form of experimental autoimmune encephalomyelitis (EAE) (Wolf et al. 1996), lipopolysaccharide (LPS)-activated B cells transferred to non-obese diabetic mice prevented the development of diabetes (Tian et al. 2001). Gut-associated lymphoid tissue-associated B cells produced IL-10 in a chronic inflammatory environment and suppressed the progression of intestinal inflammation by influencing STAT3 signaling (Mizoguchi et al. 2002). The term “regulatory B cells” was coined by Mizoguchi et al. (2002), who characterized the population of CD1d^hi^ B cells in chronic intestinal inflammatory disease (Mizoguchi et al. 2002; Mizoguchi and Bhan 2006).

The concept of Bregs playing a role of negative regulators of the immune system preventing a pathological autoreactive response and protecting from uncontrolled inflammation is now widely accepted and extensively investigated. It is considered that, both in mice and humans, Bregs suppress the immune response primarily via an IL-10-dependent mechanism (Mauri and Bosma 2012). Recently, based on studies in experimental animal models and patients with autoimmune diseases, a variety of Bregs were identified as having diverse phenotypes and mechanisms of suppression.

It was reported that B cell-deficient or lacking IL-10-producing B cells mice developed exacerbated arthritis and did not recover from experimental autoimmune encephalitis (Carter et al. 2012; Wolf et al. 1996). The exacerbation of the disease was associated with the increase in Th1 and Th17 and the decrease in Foxp3^+^ T-regulatory cells. Since IL-10 is the most common mediator of suppression, it is believed that the production of this cytokine is a characteristic marker for Breg identification.

In healthy mice, B cells with regulatory activity constitute 1–5% of spleen or lymph node B cells and up to 10% of peritoneal cavity B cells; however, their numbers increase following B cell receptor (BCR), CD40, or TLR stimulation in vitro (Fillatreau et al. 2002; Lampropoulou et al. 2008; Maseda et al. 2013; Mauri et al. 2003). In human peripheral blood, the percentage of IL-10-producing B cells amounts to 1–2% of all B cells (Blair et al. 2010). A variety of mouse and human Breg subsets are characterized by both a phenotype and mechanism of suppression, mainly IL-10-dependent (Table 2A, B).

**Table 2:** Phenotypes of regulatory B cell subsets of mouse (A) and human (B)

Regulatory B cell (mouse)	Phenotype	Mechanism of suppression	References
(A)			
B10 cells	CD19^hi^CD1d^hi^CD5^+^	IL-10	Yanaba et al. (2008, 2009)
B-1a cells	CD19^+^CD5^+^	IL-10	O’Garra et al. (1992)
MZ B cells	CD19^+^CD21^hi^CD23^−^CD24^hi^IgM^hi^IgD^lo^CD1d^hi^	IL-10	Gray et al. (2007a, b)
T2-MZP B cells	CD19^+^CD21^hi^CD23^hi^CD24^hi^IgM^hi^ IgD^hi^CD1d^hi^	IL-10	Evans et al. (2007)
GIFT-15 B cells	B220^+^CD21^+^CD22^+^CD23^+^CD24^+^ CD1d^+^CD138^+^IgD^+^IgM^+^	IL-10	Rafei et al. (2009)
Plasmablasts	CD138^+^CD44^hi^	IL-10	Matsumoto et al. (2014)
TIM-1 B cells	TIM-1^+^	IL-10	Ding et al. (2011)
Plasma cells	CD138^hi^IgM^+^TACI^+^CXCR4^+^ CD1d^hi^Tim1^int^	IL-10, IL-35	Neves et al. (2010) Shen et al. (2014)
PD-L1^hi^ B cells	CD19^+^PD-L1^hi^	PD-L1	Khan et al. (2015)
Killer B cells	CD5^+^CD178^+^	FasL	Lundy and Fox (2009)
CD73^+^ B cells	B220^+^CD39^+^CD73^+^	Adenosine	Kaku et al. (2014)

Regulatory B cell (human)	Phenotype	Mechanism of suppression	References
(B)			
B10 cells	CD19^+^CD24^hi^CD27^+^	IL-10	Iwata et al. (2011)
Immature B cells	CD19^+^CD24^hi^CD38^hi^	IL-10, PD-L1	Blair et al. (2010)
Br1 cells	CD25^hi^CD71^hi^CD73^lo^	IL-10, IgG4	van de Veen et al. (2013)
Plasmablasts	CD27^int^CD38^hi^	IL-10	Matsumoto et al. (2014)
iBregs	–	TGF-β, IDO	Nouel et al. (2015)
GrB^+^ B cells	CD19^+^CD38^+^CD1d^+^IgM^+^CD147^+^	GrB, IL-10, IDO	Lindner et al. (2013)
CD73^+^ B cells	CD39^+^CD73^+^	Adenosine	Saze et al. (2013)

A large variety of regulatory B cells have been identified. The majority of them are characterized by the production of IL-10, and the most investigated are B10 B cells detected in mice and humans

*iBregs* inducible regulatory B cells

There are still more questions than answers as regards the stability of the phenotype of Bregs. The clonotypic transcription factor or specific markers are still unknown. For instance, the majority of mouse Bregs producing IL-10 express CD5, CD1d, CD21, TIM-1 (T cell Ig domain and mucin domain protein) in combination or separately. Recently, the most investigated IL-10-producing Bregs are B10 cells, characterized by CD1d^+^CD5^+^ phenotype, which represent 1–2% of splenocytes in mice (Yanaba et al. 2008, 2009). It is widely accepted that B cell activating factor belonging to the TNF family (BAFF) is a key regulator for B cell maturation and function; however, it remains unknown whether it plays a role in regulating the development of regulatory B10 cells as well as in inducing the suppressive activity. It was shown that BAFF increased the production of IL-10 in vitro by mouse splenic B cells. BAFF-induced B cells were of the CD1d^hi^CD5^+^ phenotype and derived from marginal zone B cells. In addition, BAFF treatment in vivo increased IL-10-producing B cells in the splenic marginal zone (Yang et al. 2010). B10 cells generated upon BAFF treatment suppressed the proliferation of target CD4^+^ T cells and the production of Th1 cytokines. Currently, it is rather believed that any B cell can acquire either effector or regulatory functions pointing on the possibility that neither effector nor Breg cells are not a terminally differentiated state (Matsumoto et al. 2014; van de Veen et al. 2013).

### Origin and Development of IL-10-Producing Breg Cells

**Fig. 3:**
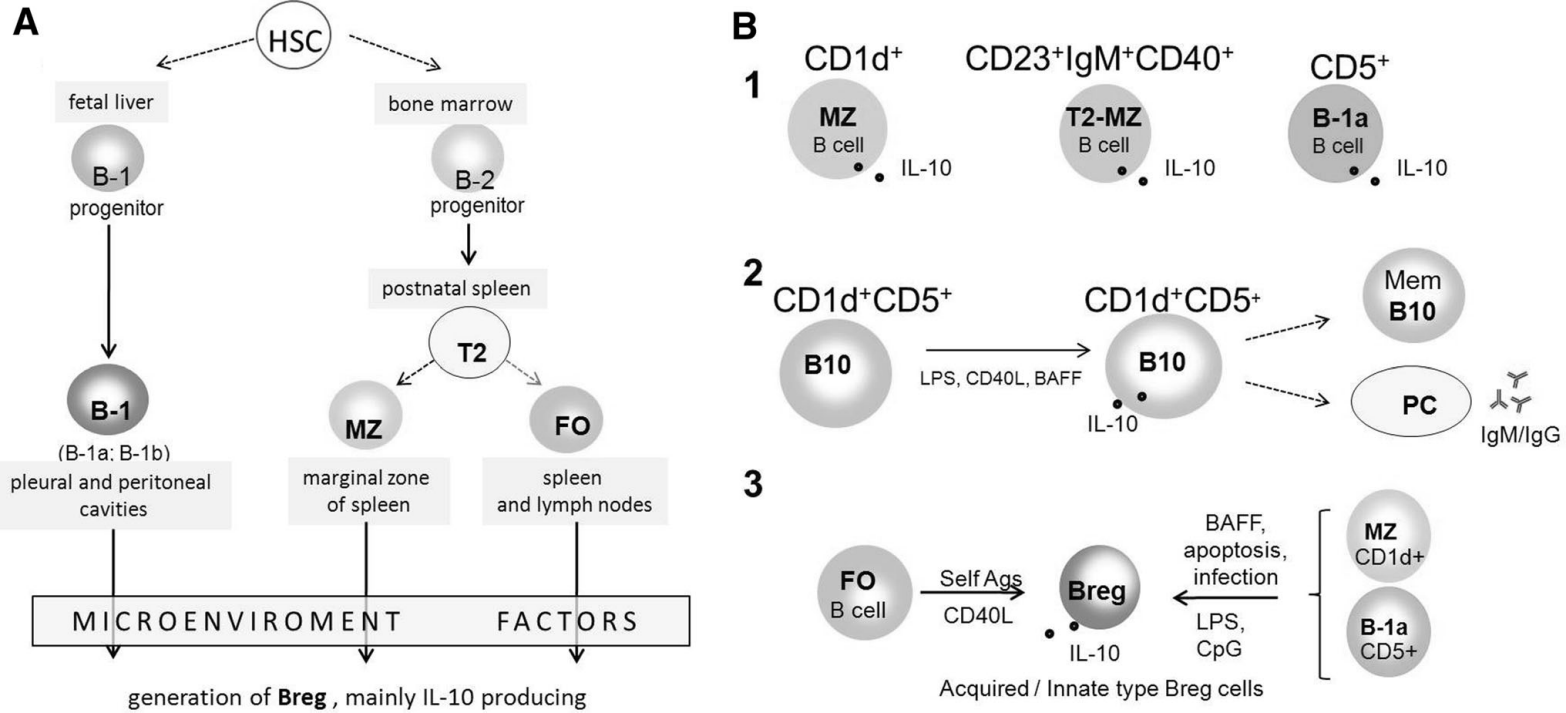
B cell development (**a**) and hypothetical models for the generation of regulatory B cells (**b**) (DiLillo et al. 2010; Gray and Gray 2010; Mizoguchi and Bhan 2006; Montecino-Rodriguez and Dorshkind 2012)**. a** B cells develop from progenitors derived from hematopoietic stem cells (HSC). B1 B cells develop from B1 progenitors in the fetal liver with little input from bone marrow beyond the perinatal period. B2 B cells arise from B2 progenitors developing into transitional 2 (T2) B cells. Next, T2 B cells differentiate into marginal zone (MZ) and follicular (FO) B cells occurring in the spleen. Weak B cell receptor (BCR) signals drive the differentiation to MZ B cells, while stronger BCR signals support the differentiation to FO B cells. **b** B cells of each lineage can develop into Bregs mainly IL-10 producing. *1* Marginal zone (MZ) B cells, transitional stage 2 marginal zone (T2-MZ) B cells, and B-1a cells contain natural regulatory B cells ready to produce IL-10 depending on microenvironmental conditions; *2* B10 cells of CD1d^+^CD5^+^ phenotype, mainly spleen-deriving, produce and secrete IL-10 upon LPS, CD40L, and BAFF stimulation. They can also differentiate into memory and/or plasma cells (indicated by *dash lines*); *3* two types of regulatory B cells producing IL-10 are postulated. “Acquired type” Breg cells are generated from follicular (FO) B cells upon stimulation by self-antigens or through CD40 signaling after CD40L ligation. “Innate type” Breg cells, which differentiate in mesenteric lymph nodes and share the phenotype of marginal zone (MZ) B cells or B-1a cells upon stimulation by BAFF or TLR ligands (LPS, CpG)

In all mammals, B cells develop in the bone marrow from hematopoietic stem cells. Here, it is important to remind the very early stage of B cell development pointing to the two lines of progenitors, which differentiate to B-1 and B-2 cells (Montecino-Rodriguez and Dorshkind 2012). A simplified pathway of B cell development is presented in Fig. 3 followed by hypothetical models of Breg cell generation.

Immature B cells (CD19^+^CD10^+^IgM^+^) from the bone marrow enter the spleen as transitional B cells and complete their development upon antigen activation as marginal zone B cells (MZ B cells) developing into short-lived plasma cells, or follicular B cells (FO B cells) developing in germinal centers into memory B cells or long-lived plasma cells (Pieper et al. 2013). Bone marrow deriving MZ and FO B cells consist of about 95% of splenic B cells referring to the B-2 subset. The remaining 5% are B-1 cells, which are deriving from both fetal liver and bone marrow, and their pool in adult mice is mainly maintained by self-renewal. The peritoneal cavity is populated mainly by B-1 cells classified as B-1a and B-1b (Barr et al. 2012; Dil and Marshall 2009). It is constantly emphasized that both mouse and human regulatory B cells are strongly heterogenous, and it is largely unknown whether they develop from a committed precursor or are induced upon particular stimulation. There are proposed 3 hypothetical models of Breg development based on their expanding diversity: (1) multi-lineage Bregs, assuming that different subsets of regulatory B cells develop from individual progenitors; (2) single-lineage Bregs, assuming that different subsets derive from a single progenitor and are characterized by a single transcription factor. Thus, at least two progenitors (B-2 and B-1 progenitors) give rise to various Bregs; (3) induced Bregs model, wherein any B cell can differentiate into Breg depending on specific microenvironmental stimuli (Mauri and Menon 2015). The latter hypothesis is strongly supported by the induction of different Bregs under different content of cytokines (Rosser et al. 2014; Wang et al. 2014). It is also possible that any B cell can differentiate into an effector or regulatory cell depending on the microenvironment created according to immunological requirements. Three different models of regulatory B cell generation are currently considered based on the involvement of microenvironmental factors (Fig. 3):

1. At least three B cell subsets, namely B-1a cells, MZ B cells, and T2-MZP contain natural Breg cells, which can produce suppressive cytokines under a certain environment (Gray and Gray 2010). Since Bregs did not have a unique phenotype or a panel of characteristic transcription factors, they can be identified by IL-10 production and suppressive function.
2. B10 cells constitute the main subset of splenic B cells, producing IL-10 (DiLillo et al. 2010; Yanaba et al. 2008). They can develop from B10 progenitor cells under LPS, CpG, or BAFF stimulation.
3. There are two types of Bregs depending on the activation pathways: “acquired” or “innate” type (Mizoguchi and Bhan 2006). The differentiation of acquired Bregs requires BCR/self-antigens binding and CD40/CD40L interaction. For the generation of innate Bregs, a polyclonal activation is necessary involving TLR ligands (LPS, CpG), apoptotic signals, or BAFF stimulation.

The role of microenvironmental factors in the induction of Bregs was confirmed in many studies. TLR agonists have been shown to induce Bregs and ameliorate autoimmune diseases. Mice with B cell deletion of TLR2 and TLR4 or MyD88 cannot recover from EAE (Lampropoulou et al. 2008). A two-step model is also considered for the acquirement of B cell-mediated suppression. The initialization of IL-10 synthesis is induced by TLR stimulation and is followed by BCR and CD40 engagement resulting in survival, expansion, and vigorous IL-10 production (Fillatreau et al. 2002).

Molecules involved in the generation and function of Breg did not differ from those engaged in the differentiation of conventional effector B cells. Activation signals are crucial to drive different functions of B cells. TLRs, which are pivotal in host defense and autoimmune response, can mediate protection in autoimmunity. Agonistic stimulation through TLR4 or TLR9 resulted in the decrease in diabetes, EAE and arthritis symptoms in mice, and the decrease in the expression of TLR9 in humans which led to increased morbidity to SLE (Buenafe and Bourdette 2007; Christensen et al. 2006; Lampropoulou et al. 2008; Quintana et al. 2000). In multiple sclerosis patients, helminths-induced CD1d^hi^ B cells produced IL-10 and inhibited the symptoms of the disease (Correale et al. 2008). Cytokine production is induced by a variety of TLR agonistic ligands, and different subsets of B cells release various sets of cytokines. Follicular B cells, upon stimulation by TLR2 or TLR4 ligands, synthesized IL-6 and IFN-γ, while MZ B cells released mainly IL-10 (Gray et al. 2007a; Lampropoulou et al. 2008). The role of TLR in the differentiation of Bregs producing IL-10 can be strengthened by CD40 delivered signal (Gray et al. 2007a; Lampropoulou et al. 2008) or concomitant in vitro activation by phorbol myristate acetate (PMA) and ionomycin (Madan et al. 2009). Bregs generated in such conditions are of the CD1d^hi^CD5^+^ phenotype. All these results showing that Bregs share similar molecules to conventional B cells confirm the hypothesis that microenvironmental factors in the body tissues drive and regulate the differentiation of various Bregs.

In general, CD40–CD40L interaction between B and T cells induces the generation of antibody-producing cells. In contrast, the prolongation or straightening of this signal inhibits the differentiation of plasma cells leading mainly to the generation of Breg cells. The role of CD40 in the induction of Bregs was documented by the experiments using mice with B cell-restricted deficiency of this molecule. These mice suffered a severe form of EAE manifested by the decrease of IL-10 production and increase of Th1- and Th17-related responses (Fillatreau et al. 2002; Mizoguchi et al. 2000). It was evidenced that in vivo administration of agonistic anti-CD40 antibodies resulted in the augmentation of Bregs subset providing IL-10 and amelioration or prevention of arthritis via inhibition of the Th1 response (Evans et al. 2007; Mauri et al. 2000, 2003). Similar therapy was also effective in lupus treatment in MRL/lpr mice (Blair et al. 2009). It was suggested that the generation of Bregs requires BCR signaling in addition to TLR and CD40 stimulation. This hypothesis is based on the fact that mice lacking CD19 (co-receptor molecule that is a member of BCR complex) developed a severe form of EAE (Sato et al. 1996). However, contradicting results are reported demonstrating an increase in IL-10-producing B cells in NFATc1-deficient mice, which is a transcription factor upregulated upon BCR stimulation (Bhattacharyya et al. 2011). Finally, it was found that the expression of costimulatory molecules, CD80 and CD86 on B cells was important in the recovery of EAE with major role of CD86 via the mechanism of regulation of regulatory T cells of the CD4^+^CD25^+^ phenotype (Mann et al. 2007).

It is already known that a small population of B cells resides in the cortical-medullary junction of the thymus where they possibly play a role in thymocyte-negative selection (Akashi et al. 2000). Recently, a population of IL-10-producing thymic B cells of the CD19^+^CD5^+^CD1d^high^ phenotype was detected and characterized by the activity to reduce the number of SP CD4^+^CD8^−^ and CD8^+^CD4^−^ thymocytes and suppress the autoimmune response in lupus-like mice (Xing et al. 2015). This indicates the thymic origin of Bregs in addition to the still poorly known pathways of their development in the periphery.

### Suppressive Mechanisms of Breg cells

Despite the phenotypical differences of Breg cells, mainly those producing IL-10, exert a suppressive function pointing on the crucial role of this cytokine (Saraiva and O’Garra 2010). Chimeric mice with B cell restricted IL-10 deficiency develop a non-remitting EAE (Fillatreau et al. 2002).

#### IL-10-Dependent Suppression

**Fig. 4:**
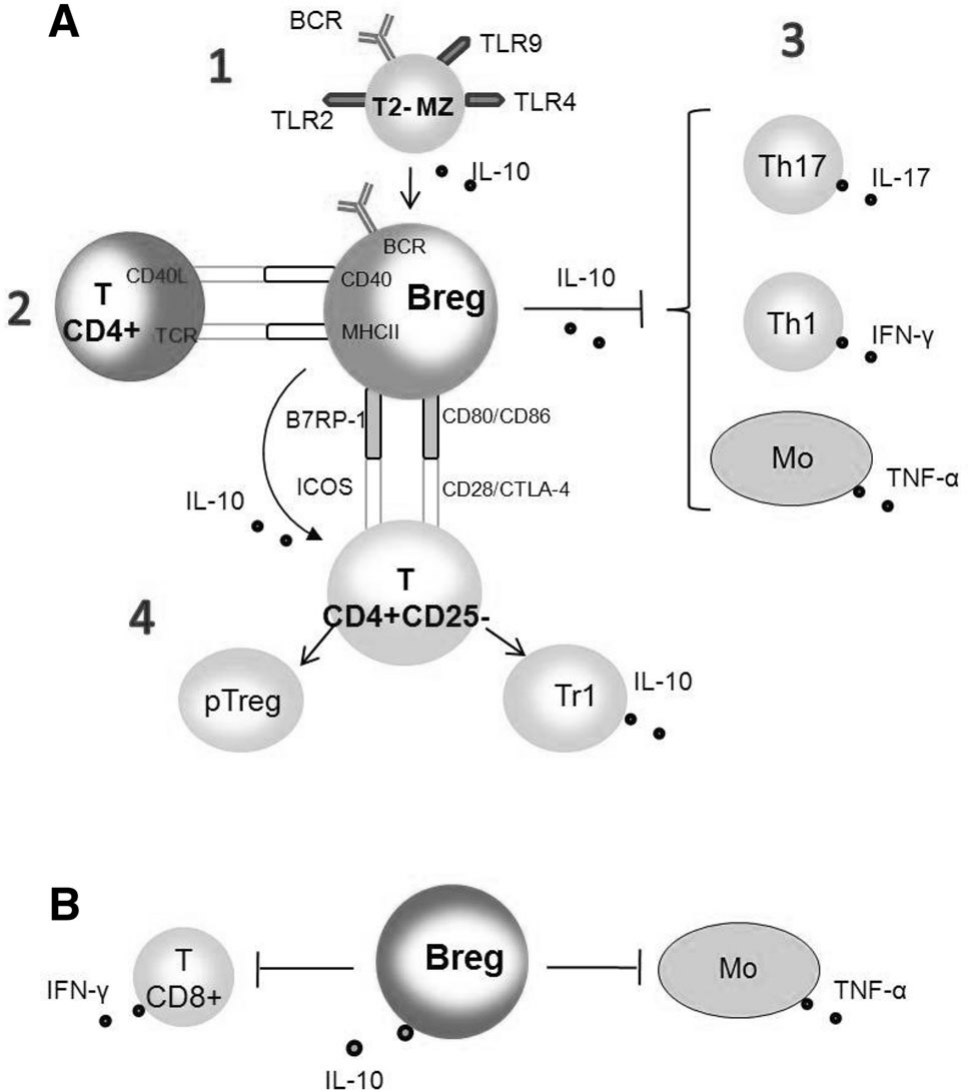
Breg-mediated IL-10-dependent suppression mechanism (Chesneau et al. 2013; Mauri and Bosma 2012). **a** Activation of potentially autoreactive T2-MZ B cells by TLR ligands originated from pathogens induces the synthesis of IL-10 (*1*). These B cells differentiate to fully active IL-10-producing Bregs upon contact with autoreactive CD4^+^ T cell and CD40/CD40L ligation. In this step, the antigen recognition by BCR is necessary (*2*). IL-10 released by Bregs suppress Th1, Th17 responses, and TNF-α production by monocytes (*3*). Interactions of Bregs with naïve T cells CD4^+^CD25^−^ involving B7RP-1/ICOS or CD80/CD86 with their ligands CD28/CTLA-4 induce the differentiation into pTregs and Tr1 regulatory cells (*4*). **b** IL-10-producing Bregs inhibit the synthesis of IFN-γ by cytotoxic T CD8^+^ cells, and TNF-α by monocytes resulting in the inhibition of anti-tumor responses. *Mo* monocytes

A variety of IL-10-dependent suppressive mechanisms can be used by Bregs (Fig. 4). Based on hypothetical models of Breg development, we can assume that they can arise from T2-marginal zone progenitor (T2-MZP) B cells sensitive to microenvironmental factors and revealing autoreactive potential (Su et al. 2004). During the response to pathogens, these potentially autoreactive T2-MZP B cells can be activated through TLRs (mainly TLR2, TLR4, TLR9) and induced to produce IL-10. Along the progression of inflammation, Bregs, upon receiving activatory signals, via BCR, CD40, and CD86/CD80, upregulate the production of IL-10. In IL-10-conditioned microenvironment, the synthesis of cytokines by Th1, Th17 cells, and monocytes can be suppressed or/and Treg cells can be generated (pTreg or Tr1) and involved in the inhibition of autoimmune diseases. Developed Bregs can suppress the synthesis of IFN-γ by CD8^+^ T cells in IL-10-dependent mechanism, which results in the inhibition of anti-tumor immunity.

IL-10-dependent mechanisms of suppression are related to the inhibition of chemokines and pro-inflammatory cytokines production, and in the downregulation of costimulatory molecules by APCs (Moore et al. 2001).

#### IL-10-Independent Mechanisms of Suppression

**Fig. 5:**
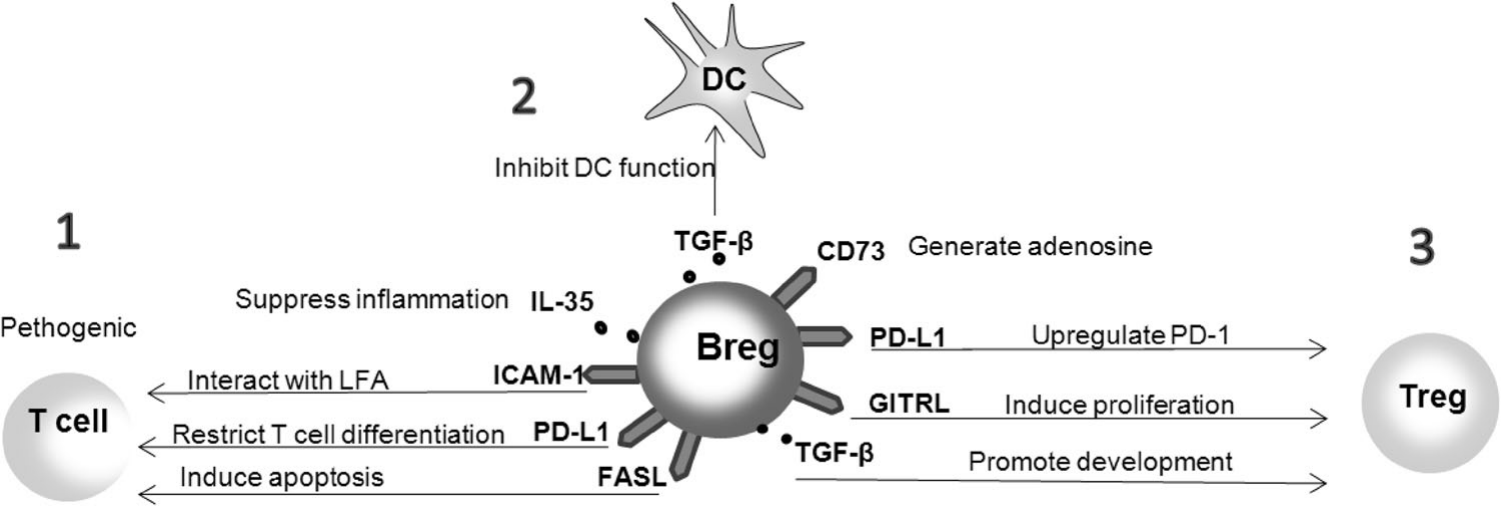
Breg-mediated IL-10-independent suppression mechanism(Ray et al. 2015). *1* Direct suppression of pathogenic T cells through IL-35, ICAM-1/LFA, FasL/Fas or PD-L1/PD-1 interactions. *2* Inhibition of the function of dendritic cells by TGF-β. *3* Inhibition of various immune cells functions through the induction of Treg development or modulation of Treg suppressive activity. *ICAM-1* intercellular adhesion molecule-1, *LFA* lymphocyte function-associated antigen, *FasL* Fas Ligand, *PD-L1* programmed death-ligand 1, *TGF-β* transforming growth factor β, *GITRL* glucocorticoid-induced TNF receptor ligand

Although the production of IL-10 is believed to be a hallmark of regulatory B cell function, other mechanisms of suppression, independent of IL-10, have been also taken into consideration (Fig. 5). Bregs can suppress the function of pathogenic T cells via IL-35, ICAM-1/LFA-1, or FasL (Fas ligand) (Minagawa et al. 2004; Shen et al. 2014). Ligation of PD-L1 on Breg and PD-1 on pathogenic T cell inhibits the proliferation and activity of pathogenic T cells (Sharpe et al. 2007). The generation of TGF-α by Bregs is thought to inhibit the function of DCs and induce the generation of Tregs (Lee et al. 2014). Bregs can expand Tregs by inducing their proliferation through GITRL/GITR interaction (Ray et al. 2012). Blockade of GITRL on B cells resulted in inhibition to maintain peripheral Tregs. PD-L1 on Bregs induce an overexpression of PD-1 on Tregs promoting their stability and expansion (Francisco et al. 2009); however, an inhibitory effect via PD-L1 ligation on Treg expansion was also documented (Franceschini et al. 2009). Bregs have been shown to generate adenosine, which plays an anti-inflammatory role in contrast to extracellular ATP. Bregs express ectoenzymes CD39 and CD73, which are the major nucleotide-metabolizing enzymes. CD39 catalyzes the breakdown of extracellular ATP to ADP and AMP followed by CD73-involved conversion of AMP to adenosine (Kaku et al. 2014). All of the IL-10-independent mechanisms of suppression are currently investigated due to the increasing interest in immunotherapies targeting B cells (Ray et al. 2015). Comparing the regulatory function of different subsets of regulatory B cells, we may conclude that their IL-10-dependent activity mainly consists in the inhibition of effector T cells or monocytes by limiting the synthesis of particular cytokines, and in inducing the differentiation of regulatory T cells, while the IL-10 independent suppression is rather related to the expansion of existing Tregs, induction of new Tregs, and, to a lesser extent, to direct effect on target pathogenic T cells.

## Conclusions

A variety of regulatory T and B cells were identified by phenotypes and particular mechanisms of action. It is widely accepted that they are involved in the maintenance of immune homeostasis and tolerance to self and foreign antigens. They can be of natural origin (not definitively confirmed for Bregs) or can be induced in peripheral lymphoid organs and tissues. In addition, they use similar mechanisms of suppression towards target cells. There is a growing number of data confirming the plasticity of regulatory T and B cells and the lack of suppressive function, despite their suppressive phenotype and specific transcription factors expression, especially in the case of Tregs. The hypothesis on the critical role of the microenvironment in the generation of regulatory cells is increasingly accepted, and on epigenetic changes in defining cell lineage stability in the context of the suppressive function. Joint efforts of many scientists are currently directed at defining a regulatory cell-type epigenetic pattern, depending on particular microenvironment enabling to obtain stable regulatory cells for clinical use.
